# Supplementary material for: Asymmetric Synthesis of Quaternary Hydantoins via a Palladium-Catalyzed Aza-Heck Cyclization
Source: J Am Chem Soc. 2025 Nov 14;147(49):44692–8. doi: 10.1021/jacs.5c16022 (PMC12703750; doi:10.1021/jacs.5c16022)
Supplement: Supplementary file 2 [file ja5c16022_si_002.zip › All NMR FID Files/S8 2D Spectra/S8 Conformer 2 HSQC/6/pdata/1/email_EM06-035-isomer#2-1HSQC_6_1.pdf]

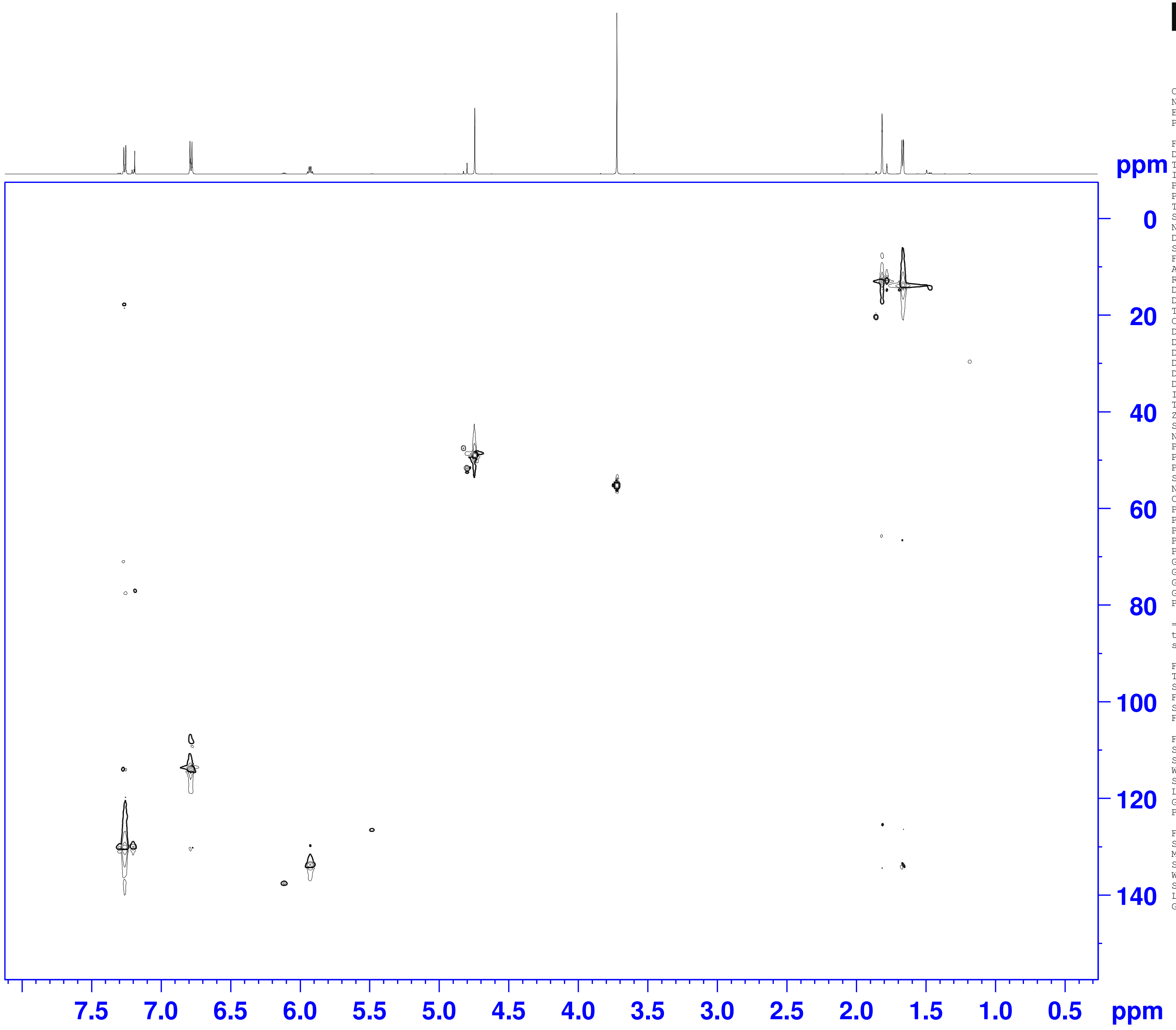


```
Current Data Parameters
NAME      EM06-035-isomer#2-1HSQC
EXPNO                    6
PROCNO                   1

F2 - Acquisition Parameters
Date_             20250211
Time                 15.40 h
INSTRUM  Avance Neo 600 at University of Delaware
PROBHD   Z185170_0001 (
PULPROG      hsqcedetgp
TD                    1024
SOLVENT              CDCl3
NS                       2
DS                      16
SWH               4716.981 Hz
FIDRES            9.212853 Hz
AQ               0.1085440 sec
RG                     101
DW                 106.000 usec
DE                   10.00 usec
TE                   298.0 K
CNST2          145.0000000
D0              0.00000300 sec
D1              1.50000000 sec
D4              0.00172414 sec
D11             0.03000000 sec
D16             0.00020000 sec
D21             0.00345000 sec
IN0             0.00002009 sec
TDav                     1
ZGOPTNS
SFO1           600.1325742 MHz
NUC1                    1H
P1                    8.07 usec
P2                   16.14 usec
PLW1            6.90000010 W
SFO2           150.9141262 MHz
NUC2                   13C
CPDPRG[2              garp
P3                   12.55 usec
P4                   25.10 usec
PCPD2                60.00 usec
PLW2          106.00000000 W
PLW12           4.63759995 W
GPNAM[1]       SMSQ10.100
GPZ1                 80.00 %
GPNAM[2]       SMSQ10.100
GPZ2                 20.10 %
P16                1000.00 usec

======== F1 INDIRECT DIMENSION ========
td1      256
sw_F1    165.000000

F1 - Acquisition parameters
TD                     256
SFO1              150.9141 MHz
FIDRES          194.537735 Hz
SW                 165.000 ppm
FnMODE        Echo-Antiecho

F2 - Processing parameters
SI                    1024
SF             600.1300578 MHz
WDW                  QSINE
SSB                      2
LB                       0 Hz
GB                       0
PC                    1.40

F1 - Processing parameters
SI                    1024
MC2          echo-antiecho
SF             150.9028085 MHz
WDW                  QSINE
SSB                      2
LB                       0 Hz
GB                       0
```
